# Cutaneous Squamous Cell Carcinoma on the Eyebrow of a Woman: A Rare Case Report

**DOI:** 10.7759/cureus.65447

**Published:** 2024-07-26

**Authors:** Omed S Hamamin, Farman U Shareef, Halkawt B Ahmed, Sattar I Kareem, Jeza M Abdul Aziz

**Affiliations:** 1 Dentistry, Komar University of Science and Technology, Sulaymaniyah, IRQ; 2 Medical Laboratory Science, Charmo University, Sulaymaniyah, IRQ; 3 Baxshin Research Center, Baxshin Hospital, Sulaymaniyah, IRQ; 4 Dermatology, Baxshin Hospital, Sulaymaniyah, IRQ; 5 Medical Laboratory Science, University of Human Development, Sulaymaniyah, IRQ; 6 Biomedical Sciences, Komar University of Science and Technology, Sulaymaniyah, IRQ

**Keywords:** elderly population, case report, eyebrow, non-melanoma skin cancer, cutaneous squamous cell carcinoma

## Abstract

The exact incidence of cutaneous squamous cell carcinoma (CSCC) or nonmelanoma skin cancer is unknown, and it is believed that the rate of occurrence is increasing with the growing elderly population and sun exposure, and it is more prevalent in males than in females. In this article, we describe the case of an 81-year-old woman who presented with a lesion on the right upper eyebrow. The patient had been consulting a dermatologist and undergoing treatment for three months. However, the lesion did not show any signs of improvement, and the dermatologist speculated that it might be a common wound that was healing slowly because of her diabetes. Imaging revealed an ulcerating skin lesion on the right upper eyebrow without connection to the deeper structures. Surgical intervention was chosen with the patient’s consent. This rare case of CSCC on a woman's eyebrow showed that skin cancer can occur in unusual locations and in people without risk factors.

## Introduction

Cutaneous squamous cell carcinoma (CSCC), or keratinocyte carcinoma, is the second most common type of nonmelanoma skin cancer, accounting for 20% of cutaneous malignancies [1], with an estimated death of more than 15,000 people in the United States in one year [2]. This is true for Caucasians, with approximately one million cases annually [3].

Nonmelanoma skin cancer has two main subtypes: CSCC and basal cell carcinoma (BCC) with incidences of 25% and 75%, respectively [4]. The number of newly recorded cases of these subtypes continues to increase [1,5]. CSCC arises from the malignant proliferation of epidermal keratinocytes [6]. The exact incidence of this type of keratinocyte skin cancer is unknown, but it is believed that the rate of occurrence is increasing with the growing elderly population [6]. The factors that play a role in the occurrence of CSCC are multifactorial, including a combination of environmental (cumulative lifetime sun exposure), immunological (organ transplant recipients), and genetic factors [7]. Additional risk factors that seem to contribute to CSCC development include the use of tanning beds, especially early in life (<25 years), beta-human papillomavirus (HPV) infection, smoking, human immunodeficiency virus (HIV) infection, hematopoietic stem cell transplantation, and long-term cutaneous inflammation such as chronic wounds, burns, scars, ulcers, or sinus tracts [1].

Depending on the lifestyle, skin type, and age of the person, squamous cell carcinoma (SCC) might develop in sun-exposed areas, such as the frontotemporal and nasal regions, as well as bald male scalps [8]. SCC progresses slowly and has scaly, painless, erythematous patches, nodules, or sharp plaque boundaries. Generally, some skin conditions are differential diagnoses of SCC, making biopsies critical for diagnosis [9,10]. We describe the case of an 81-year-old female who presented with an ulcerated lesion on the right upper eyebrow.

## Case presentation

An 81-year-old woman with diabetes and hypertension presented to the hospital with a history of a lesion on the lateral border of the right upper eyebrow. The patient had been visiting a dermatologist and receiving treatment for three months. Still, the lesion did not improve, as the dermatologist thought it may be a usual wound with delayed healing due to her diabetes.

**Figure 1:**
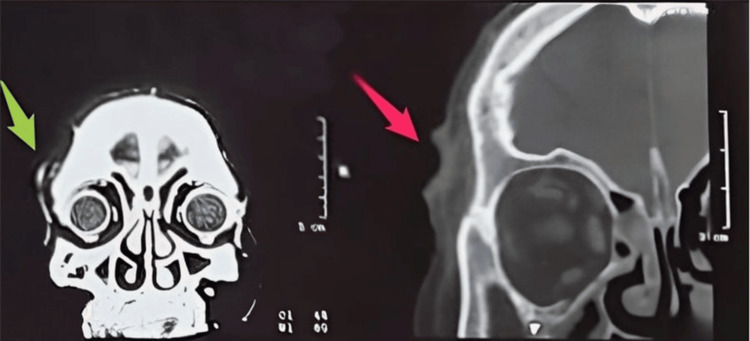
Ulcerating skin lesion seen on the right upper eyebrow (red arrow) with no connection to deeper structures (green arrow)

The patient had no personal or family history of skin cancer. She denied a history of smoking or alcohol consumption and had no known allergies to any medication. She did not have an outdoor occupation and was a housewife. An ulcerated lesion was observed on the right upper eyebrow. The lesion was suspected to be BCC or SCC. The patient was referred for a computerized tomography (CT) scan with contrast to assess the depth and tissue invasion of the lesion as well as metastasis to the bone or brain. Imaging reported that the lesion was a 16 × 7 mm enhancing ulcerating skin lesion seen on the right upper eyebrow with no connection to deeper structures (Figure 1).

**Figure 2:**
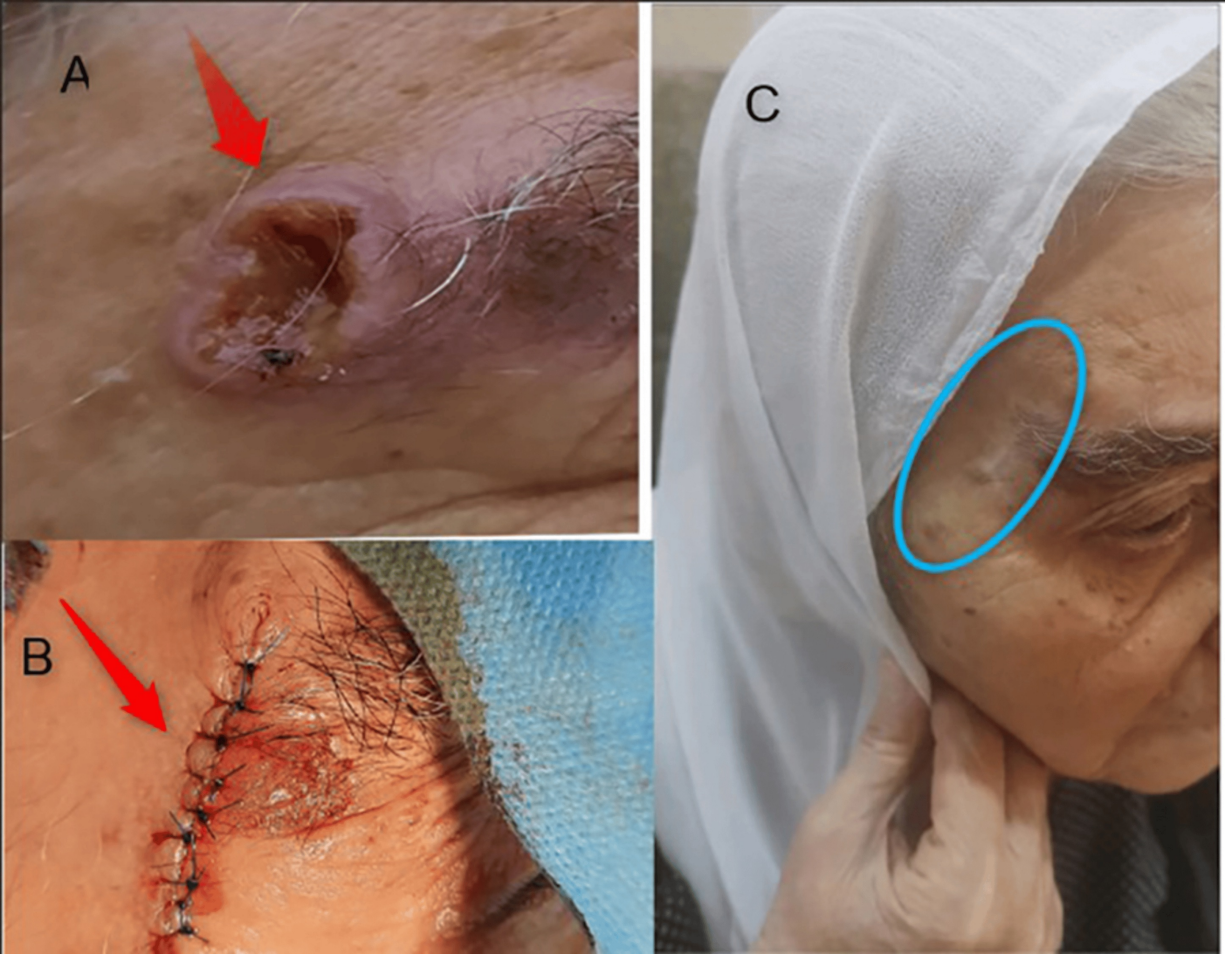
(A) Ulcerated squamous cell carcinoma lesion on the right upper eyebrow. (B) The lesion site after suturing. (C) The lesion site one year after the procedure.

Based on the high suspicion of malignancy and imaging results of an ulcerated lesion on the right upper eyebrow (Figure 2, Panel A), surgical intervention was decided upon with the patient’s consent. The patient's suitability for general anesthesia was assessed using an echocardiogram. The procedure was performed under general anesthesia. An incision was made to remove the lesion with a 4 mm margin of normal skin using a No. 10 blade, and cauterization was performed for the bleeding. Irrigation and debridement were performed on the wound, then sutured (Figure 2, Panel B). The specimens were placed in formalin and sent for histopathological evaluation. Following the procedure, the patient was prescribed oral antibiotics (ciprofloxacin tablets) and paracetamol tablets (1 g). Fucidin ointment was also prescribed for the first three days and applied three times a day. After three days, the patient used MEBO Scar ointment persistently. Every three months, the patient was followed up by checking the lesion site to detect any pain or undesirable sensation around the scar. One year after the diagnosis and procedure, the scar had healed very well (Figure 2, Panel C), and the patient had not reported any symptoms.

**Figure 3:**
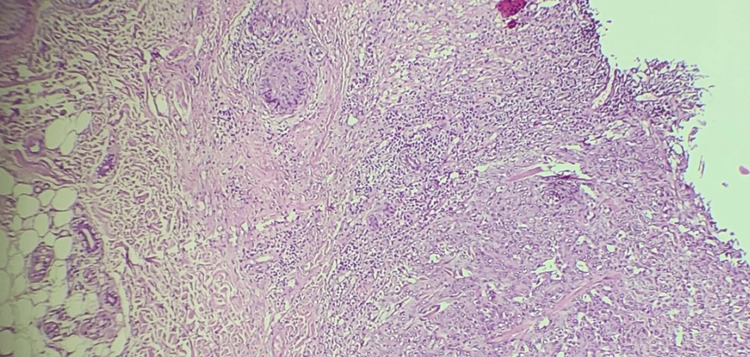
Histopathological appearance of cutaneous squamous cell carcinoma

The histopathological results showed that the lesion was a tumor with features highly suggestive of poorly differentiated SCC. The level of tumor invasion involved the reticular dermis. Lymph vascular and perineural invasion were not identified (Figure 3).

## Discussion

CSCC is the second most common skin malignancy in the world after BCC [11]. According to Kim et al., men were more likely to have CSCC arising on the head and neck (51.7%). In contrast, women were more likely to develop CSCCs on the lower extremity (41.2%) [12]. However, our case involved a female patient with CSCC at the head, particularly in the eyebrow region. The eyebrow region is an uncommon location for CSCC. Typically, CSCC arises in sun-exposed areas, such as the face, ears, scalp, and neck [13]. The rarity of this case highlights the potential for CSCC to develop at unexpected sites, emphasizing the importance of thorough examination and evaluation of any suspicious skin lesions.

Our patient was 81 years old, and aging itself increases the risk of CSCC according to Wiser et al. [13]. Although this patient did not possess typical risk factors associated with CSCC, such as chronic sun exposure, fair skin, or a history of radiation therapy, it is essential to recognize that CSCC can occur in individuals without these predisposing factors. However, the exact cause of this rare presentation remains unclear. Long-lasting chronic inflammatory processes, such as those observed in chronic wounds, may also contribute to the development of CSCC [14].

The diagnosis of CSCC is confirmed through histopathological examination, which is a crucial step in managing skin cancers [15]. Staging, often guided by imaging studies, is essential for determining the extent of the disease. In this case, contrast-enhanced CT was used to assess local invasion and the absence of distant metastasis [10]. The management of this rare case posed unique challenges. Given the location of the eyebrows, surgical excision was considered. However, preserving the aesthetic and functional aspects of the eyebrow is a primary concern. In this case, the lesion was excised with a 4 mm free margin [16].

In the early stage, soon after detection, CSCC can be treated fully with significantly decreased morbidity. Certain subtypes of CSCC may show less prognosis for patients [17]. Due to certain patient characteristics and pathological properties, recurrence could be seen (>5%), and metastasis to the lymph nodes around or to distal nodes appears in different frequencies [18,19]. Prognosis in CSCC generally depends on factors such as tumor size, depth of invasion, and presence of regional or distant metastasis [20]. In this rare case of eyebrow CSCC, early diagnosis and comprehensive treatment contributed to a favorable prognosis. The patient was regularly monitored for recurrence or metastasis, underscoring the significance of long-term follow-up in skin cancer cases [15].

## Conclusions

This extremely unusual case of CSCC on a woman's eyebrow serves as a reminder that skin cancer can manifest in unusual locations and in people who do not have traditional risk factors for the disease. To achieve the best possible outcomes in these cases, prompt diagnosis, precise staging, and individualized treatment plans are necessary. Additionally, to address the one-of-a-kind challenges presented by rare presentations of skin cancer and to maintain both esthetics and function, interprofessional collaboration among medical professionals is necessary.
